# A practical guide for performing arthrography under fluoroscopic or ultrasound guidance

**DOI:** 10.1007/s13244-015-0442-9

**Published:** 2015-10-22

**Authors:** Eugen Lungu, Thomas P Moser

**Affiliations:** Faculty of Medicine, University of Montreal, Montreal, QC Canada; Department of Radiology, Centre Hospitalier de l’Université de Montréal (CHUM), Hôpital Notre-Dame, 1560 rue Sherbrooke Est, Montréal, QC H2L 4M1 Canada

**Keywords:** Arthrography, Articular recess, Intra-articular injection, Fluoroscopy, Ultrasound

## Abstract

**Abstract:**

We propose a practical approach for performing arthrography with fluoroscopic or ultrasound guidance. Different approaches to the principal joints of the upper limb (shoulder, elbow, wrist and fingers), lower limb (hip, knee, ankle and foot) as well as the facet joints of the spine are discussed and illustrated with numerous drawings. Whenever possible, we emphasise the concept of targeting articular recesses, which offers many advantages over traditional techniques aiming at the joint space.

***Teaching Points*:**

• *Arthrography remains a foremost technique in musculoskeletal radiology*

• *Most joints can be successfully accessed by targeting the articular recess*

• *Targeting the recess offers several advantages over traditional approaches*

• *Ultrasound-guidance is now favoured over fluoroscopy and targeting the recess is equally applicable*

## Introduction

**Fig. 1:**
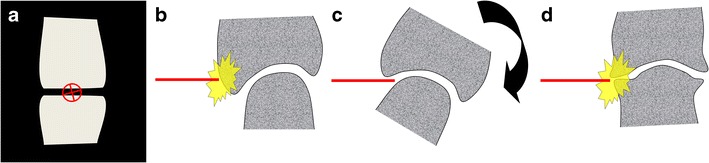
Commonly encountered difficulties with direct puncture of the radiological joint space. Direct access to the radiological joint space (**a**) may be impaired by the normal anatomy (spheroid and condyloid joints) (**b**) and could require repositioning of the limb that may, at times, be challenging (**c**). Degenerative changes such as joint space narrowing and osteophytes may also hinder a direct approach (**d**)

**Fig. 2:**
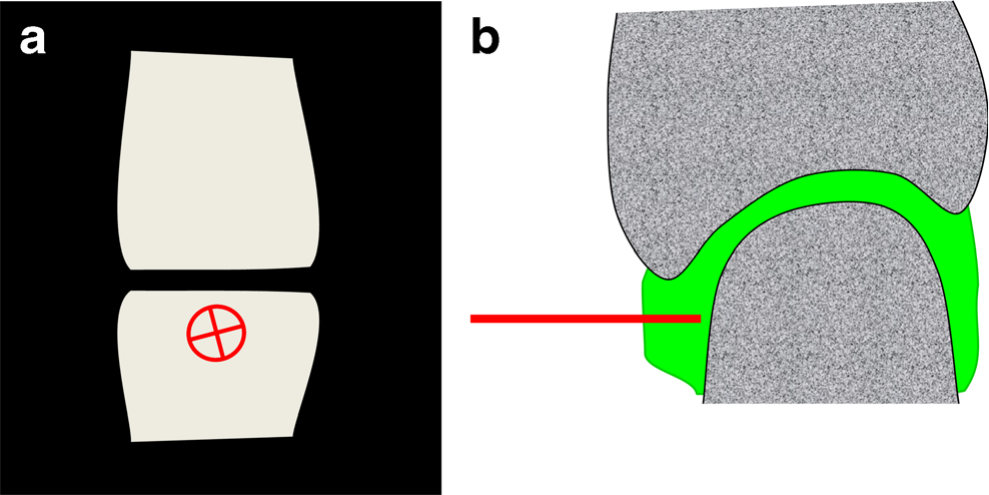
Targeting the articular recess rather than the radiological joint space on an anteroposterior radiograph (**a**) and its corresponding sagittal anatomical diagram (**b**). This approach can alleviate the difficulties mentioned previously

Arthrography has been an essential technique in musculoskeletal radiology for more than 100 years now and remains useful in combination with computer tomography and magnetic resonance imaging for a detailed assessment of articular structures, or by itself as a way to confirm the adequate distribution of therapeutic injections [1, 2]. More recently, modifications of the technique using alternative approaches such as those targeting the articular recesses [3], and/or ultrasound guidance have been published [4, 5]. Targeting the articular recess instead of the radiological joint space (Fig. 1) is optimal when the latter is not accessible due to overlapping normal bone structures or severe degenerative changes such as osteophytes (Fig. 2), and may help to avoid patient manipulation and tube angulation [3]. With this technique, the needle is advanced until contact with bone, thus providing a depth limit to insertion and potentially increasing the safety of the procedure. Moreover, this approach can help avoid articular fibrocartilages (labra and menisci). Finally, this technique is transposable to ultrasound guidance where the needle is best placed tangentially to the transducer rather than vertically. Indeed, ultrasound guidance for performing arthrography is now favoured over fluoroscopy by many specialists [6]. The principal advantages are the absence of ionising radiation for the patient and the operator, the possibility of operating ultrasound equipment outside of a radiology department (office practice), and imaging of all the soft tissues surrounding the joint, leading to an accurate diagnosis prior to the therapeutic injection and avoidance of any critical structures in the path of the needle.

In this article, we aim to review the anatomical bases and approaches for arthrography of upper and lower limbs as well as of facet joints of the spine. We do not intend to be exhaustive in the approaches described and in many instances we present those favoured by the authors. Although the descriptions are based on fluoroscopic guidance, we encourage the readers to translate this knowledge to ultrasound-guided arthrography by providing a few examples.

## General technique of arthrography

The technique varies slightly depending on the articulation and guidance modality, but can be outlined as follows:

### Positioning of the patient and the articulation

The patient is placed in a manner appropriate for the injected joint. Under fluoroscopy guidance, the optimal positioning occurs when the needle can be inserted straight along the direction of the X-ray beam without tube angulation. With ultrasound guidance, the positioning is more liberal and should be comfortable for both the patient and the operator.

### Insertion of the needle

Ideally, the tip of the needle should be superimposed over the hub on control fluoroscopic images. Upon bone contact, the needle is very slightly withdrawn and the injection is tested with an anaesthetic agent. Alternatively, the needle can be gradually inserted while exerting gentle pressure on the anaesthetic syringe plunger and intra-articular location is presumed when a decrease in resistance is felt.

With ultrasound guidance, the needle has to be introduced tangentially to the transducer in order to be adequately followed until it reaches the joint capsule.

### Confirmation of intra-articular position

Under fluoroscopic guidance, contrast medium is generally used to confirm intra-articular location. With gentle and progressive injection, flow of contrast medium away from the needle tip and opacification of the joint space confirm adequate position. For therapeutic injections of large joints in patients with allergy to contrast substances, it is possible to inject a small volume of air (3-5 ml) as a substitute.

With ultrasound guidance, the injected substance is easily seen flowing out of the needle and distending the joint capsule.

### Injected substances

For therapeutic injections, steroids are most commonly employed. Triamcinolone acetonide (40 mg/ml) or methylpredinosolone acetate (40 mg/ml) are widely used in North America at a dose ranging from 10 mg or less for small articulations to 40 mg for large articulations. In Europe, cortivazol (3.75 mg/1.5 ml) is the most frequently administered formulation. These different steroids have a tendency to form particles, which can be source of deleterious effects or complications. For this reason, it is recommended to substitute a non-particulate steroid, such as dexamethasone sodium phosphate (10 mg/ml), in specific circumstances that are beyond the scope of this paper [7]. Steroids can be mixed with a liberal amount of local anaesthetic.

For diagnostic injections, the injectate is iodinated contrast medium (at a concentration of 240 mgI/ml or less) for CT arthrography, and a dilution of 1/200 to 1/250 of gadolinium chelates in iodinated contrast medium or sterile saline (reaching a concentration of 0.0020–0.0025 mmol/ml) for MR arthrography. Although not approved by the United States Food and Drug Association, mixing iodinated contrast media with gadolinium chelates has been done for many years now in North America and other countries with no harmful effects [8]. On the other hand, ready-to-inject preparations are commercialised in most European countries and should thus be favoured.

## Upper limb

### Shoulder

**Fig. 3:**
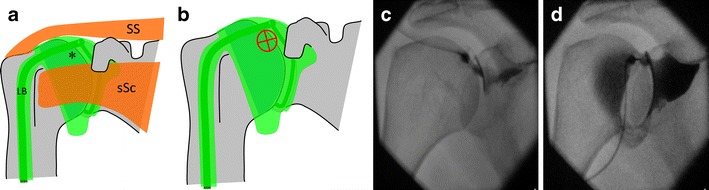
Glenohumeral joint injection by targeting the rotator interval. **a** The rotator interval (*) is limited superiorly by the supraspinatus tendon (*SS*), anteriorly by the subscapularis tendon (*sSc*) and contains the long head of the biceps tendon (*LB*). **b** The target is the upper medial quadrant of the humeral head. **c** and **d** Insertion of the needle until bone contact and confirmation of adequate position by opacification of the joint space and of the subscapularis recess

For arthrography of the glenohumeral joint, an anterior approach targeting the rotator cuff interval can be performed [9]. The steps required for this technique are the following (Fig. 3):

1. The shoulder is positioned in a straight anteroposterior view, with the arm in external rotation. The target is the upper medial quadrant of the humeral head. The coracoid process is easily avoided in this position.
2. A 1.5-inch (3.8-cm) 22-gauge needle is inserted until bone contact and the injection is tested with lidocaine.
3. Flow of contrast medium away from the needle tip and opacification of the joint space and of the subcoracoid recess confirm intra-articular positioning. The joint capacity is normally about 8-15 ml, but can be reduced to less than 7 ml in adhesive capsulitis.

**Fig. 4:**
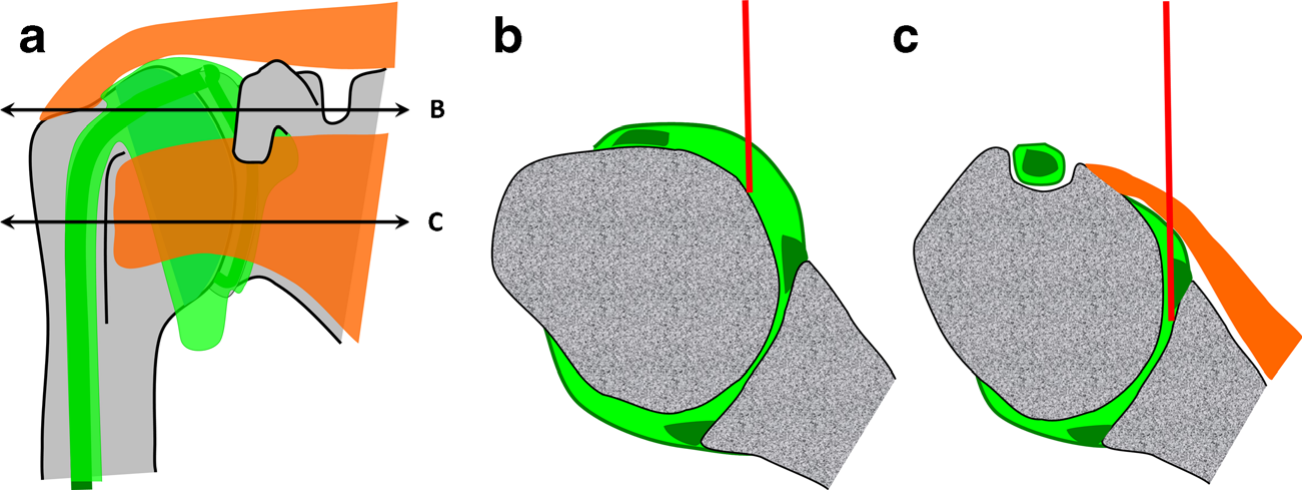
**a** Comparison of the rotator interval approach to the Schneider technique on a diagrammatic representation of the shoulder joint, with two transverse sections (*B* and *C*). **b** At the level of the rotator interval, the needle path is shorter and the long head of the biceps tendon is easily avoided by placing the arm in external rotation. **c** With the Schneider technique, the subscapularis tendon and antero-inferior labrum are regularly perforated by the needle or impregnated by the contrast agent

The advantages of the rotator interval approach compared with the very commonly used Schneider technique are the transgression of fewer anatomical structures and the use of shorter needles (Fig. 4) [10–12]. In particular, the risk of distorting the antero-inferior labrum and capsule, the inferior glenohumeral ligament and the subscapularis tendon is avoided. Positioning the patient’s shoulder in external rotation ensures sparing of the long head of the biceps.

Another approach on a prone patient is to target the posterior aspect of the humeral head. Both anterior and posterior approaches can also be performed with ultrasound guidance [5].

### Elbow

**Fig. 5:**
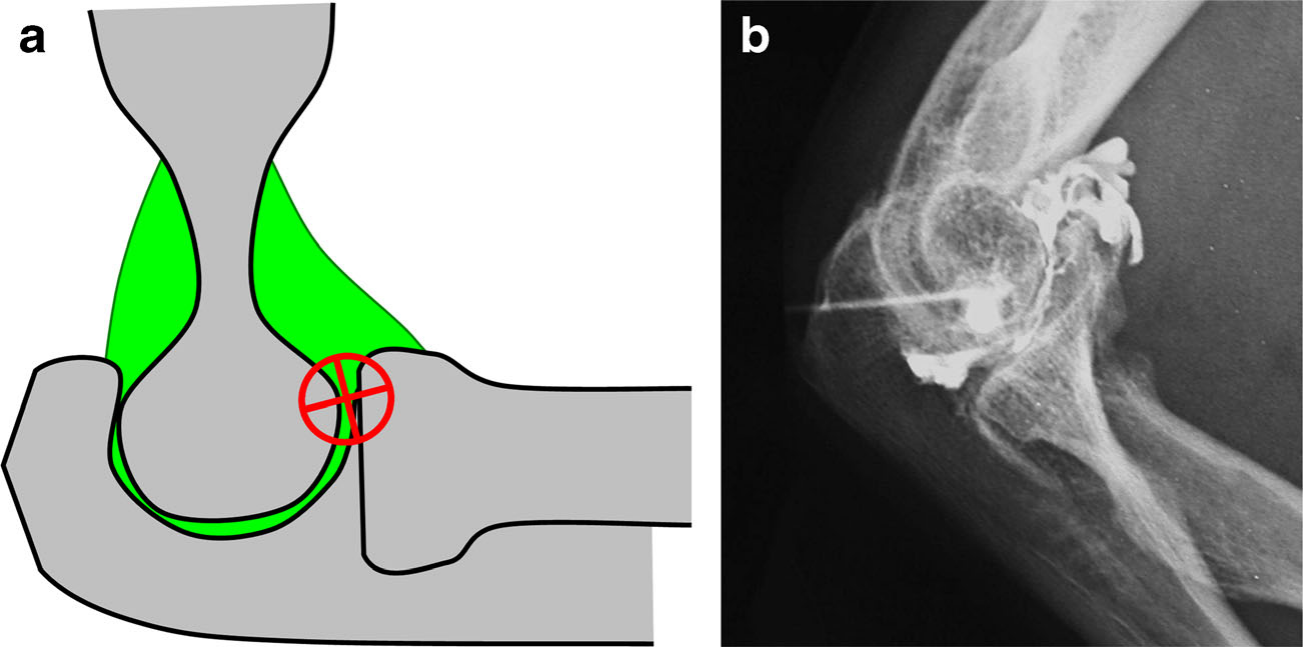
Representation of the elbow joint depicting elbow arthrography. **a** The needle is inserted in the anterior aspect of the humeroradial compartment by passing through the capsule. **b** Confirmation of adequate position by opacification of the anterior and posterior recesses

Arthrography of the elbow is commonly performed through the humeroradial compartment (Fig. 5).

1. The patient is positioned supine with their hand behind their back, exposing the lateral aspect of the elbow. The target is the anterior aspect of the humeroradial compartment.
2. A 1.5-inch (3.8-cm) 25-gauge needle connected to a syringe of lidocaine is inserted. Passing through the capsule followed by loss of resistance is easily felt.
3. Flow of contrast medium away from the needle tip and opacification of the anterior and posterior recesses confirm adequate position. The joint capacity is about 5 ml.

Alternatively, it is possible to target the olecranon fossa with a posterior transtriceps approach, which can be easily transposed to ultrasound-guided arthrography [13].

### Wrist

**Fig. 6:**
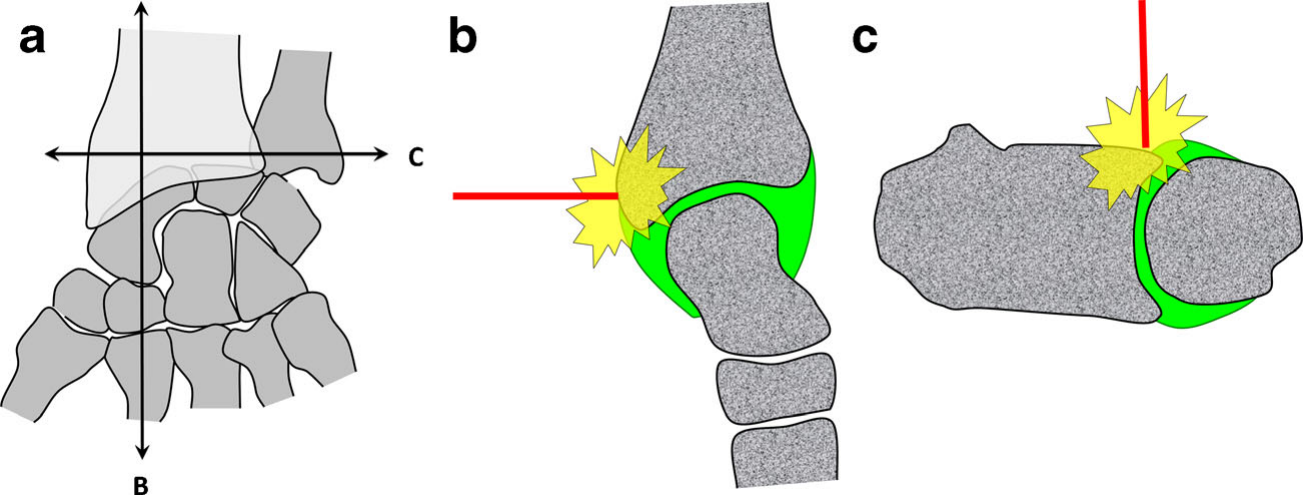
Representation of the wrist joint (**a**) with sagittal (*B*) and transverse (*C*) sections. The posterior radius overlies both radiocarpal (**b**) and distal radioulnar (**c**) joint spaces, precluding direct approaches

The wrist joint compartments are all accessed using a dorsal approach. Figure 6 shows that the posterior aspect of the distal radius overlaps both the radiocarpal and the distal radioulnar joint spaces, which can be a pitfall for a direct approach to these joint spaces.

**Fig. 7:**
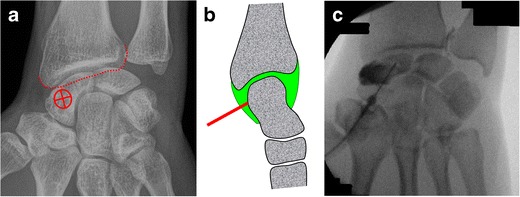
Radiocarpal joint injection by targeting the posterior radioscaphoid recess. **a** The target is the mid portion of the scaphoid. **b** The needle is angled proximally until bone contact. **c** Confirmation of adequate position by opacification of the radioscaphoid recess

Arthrography of the radiocarpal joint can be performed as follows [14] (Fig. 7):

1. The wrist of the patient is positioned prone with mild ulnar deviation to lengthen the scaphoid. The target is the midportion of the scaphoid.
2. A 1.5-inch (3.8-cm) 25-gauge needle is inserted in a slight caudocranial direction upon bone contact.
3. Flow of contrast medium away from the needle tip and opacification of the periscaphoid recess confirm adequate position. The joint capacity is about 3 ml.

**Fig. 8:**
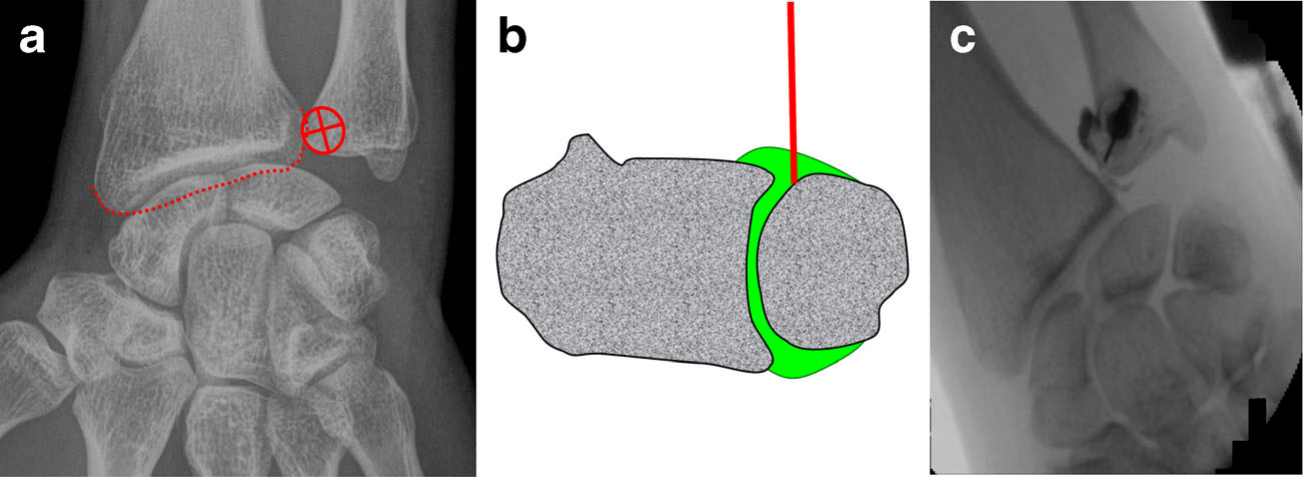
Distal radioulnar joint injection by targeting the posterior radioulnar recess. **a** The target is the distal and lateral aspect of the ulnar head. **b** The needle is inserted in a straight direction until bone contact. **c** Confirmation of adequate position by opacification of the radioulnar compartment

For distal radioulnar joint arthrography [15, 16] (Fig. 8):

1. The wrist of the patient is positioned prone. The target is the radial and distal aspect of the ulnar head.
2. A 7/8-inch (2.2-cm) 25-gauge needle is inserted until bone contact.
3. Flow of contrast media away from the needle tip and opacification of the compartment confirm adequate position. The joint capacity is about 2 ml.

When a triple-compartment opacification is required, the midcarpal joint can be accessed in the space between the lunate, triquetrum, capitate and hamate.

For ultrasound-guided injections, the posterior recesses of the radiocarpal and midcarpal joints can be targeted in both transverse and sagittal planes.

### Fingers and toes

**Fig. 9:**
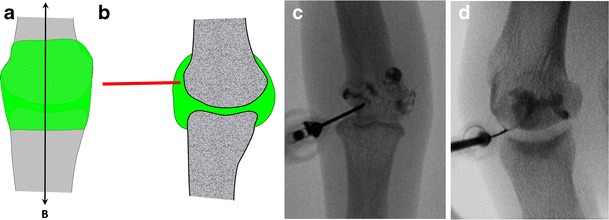
Diagrammatic representation of a metacarpophalanngeal joint (**a**) with corresponding sagittal section (**b**) showing that the articular recess is more developed on the dorsal aspect of the metacarpal head and can be easily punctured. Interphalangeal (**c**) and metatarsophalangeal (**d**) arthrographies are performed by using the same principle

Arthrography of the metacarpophalangeal, metatarsophalangeal, and interphalangeal joints can be performed by targeting the dorsal articular recess. The following steps are required (Fig. 9):

1. The hand/foot of the patient is positioned prone. The target is the distal aspect of the metacarpal/metatarsal or phalanx proximal to the joint.
2. A 5/8-inch (1.6-cm) 25-gauge needle is inserted until bone contact.
3. Flow of contrast medium away from the needle tip and opacification of the compartment confirm adequate position. The joint capacity is about 1 ml.

**Fig. 10:**
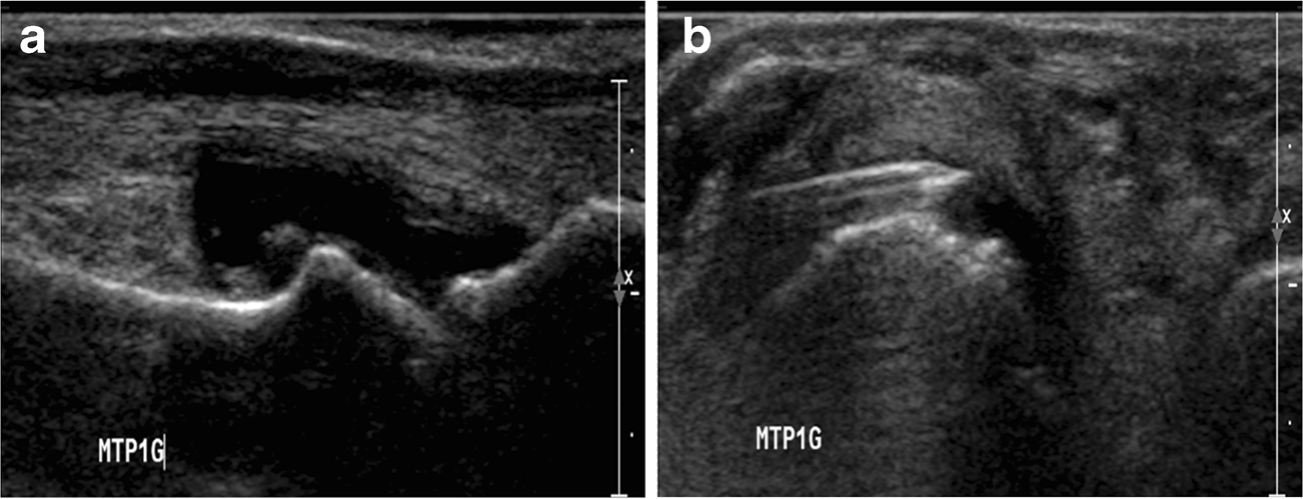
Ultrasound-guided aspiration of the first metatarsophalangeal joint. **a** Longitudinal view demonstrating distension of the dorsal recess by joint effusion. Note that the dorsal recess extends proximally on the dorsum of the first metacarpal. **b** Transverse view demonstrating the insertion of the needle in the dorsal recess and fluid aspiration

Alternatively, the dorsal recess is also accessible under ultrasound guidance (Fig. 10).

## Lower limb

### Hip

**Fig. 11:**
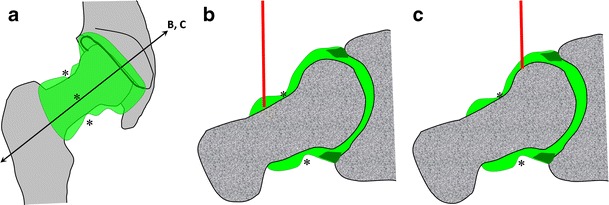
Diagrammatic representation of the hip joint (**a**) with transverse oblique sections (*B*, *C*). Both the lateral (**b**) and medial (**c**) portions separated by the zona orbicularis (*) can be targeted for hip injections

Because of the anatomical configuration of the coxofemoral joint, the approach for arthrography of the hip necessarily targets the anterior recess. The anterior recess encompasses lateral and medial portions separated by the zona orbicularis (annular ligament). Any of these portions can be targeted successfully (Fig. 11).

**Fig. 12:**
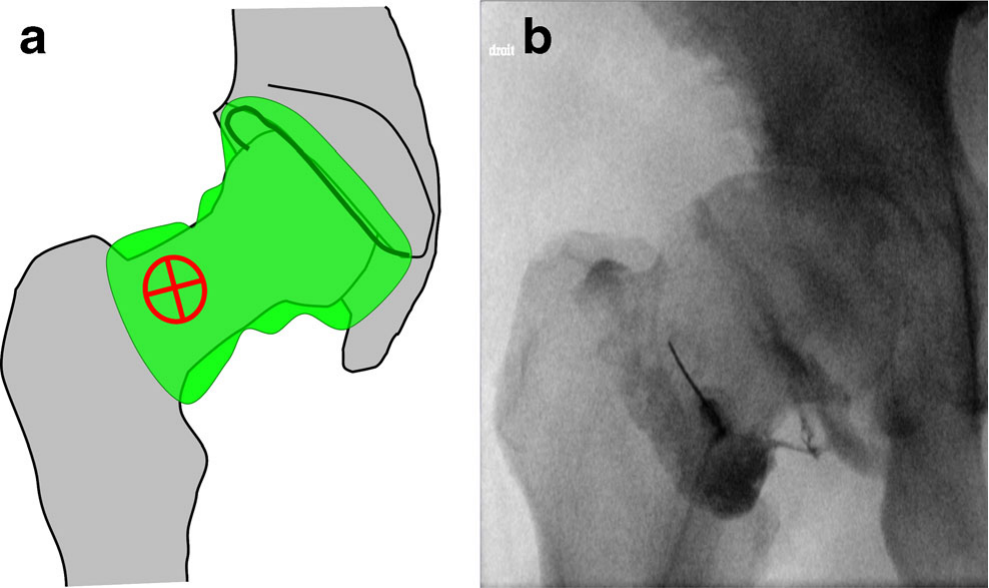
Hip joint injection by targeting the lateral portion. **a** The target is the lateral aspect of the femoral neck. **b** The needle is inserted until bone contact and opacification of the joint space confirms the adequate position

For the arthrography of the hip joint by targeting the lateral portion, the following steps are required (Fig. 12):

1. The hip of the patient is positioned in slight medial rotation. The target is the lateral aspect of the femoral neck.
2. A 3.5-inch (8.9-cm) 22-gauge needle is inserted until bone contact and the injection is tested with lidocaine.
3. Flow of contrast medium away from the needle tip and opacification of the joint space confirm intra-articular positioning. The joint capacity is about 10 ml.

This approach was shown to be less painful than the medial approach, but was associated with greater extra-articular contrast leakage in one study [17]. In our experience, this lateral approach is advantageous in obese patients, where the medial approach can be jeopardised by the overlying belly.

**Fig. 13:**
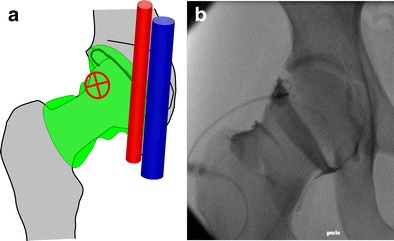
Hip joint injection by targeting the medial portion. **a** The target is the superior aspect of the head-neck junction away from the femoral vessels. **b** The needle is inserted upon bone contact and opacification of the joint space confirms the adequate position

The arthrography of the hip joint by targeting the medial portion can be performed as follows (Fig. 13):

1. The hip of the patient is positioned in slight medial rotation. The target is the superior head-neck junction.
2. A 3.5-inch (8.9-cm) 22-gauge needle is inserted until bone contact and the injection is tested with an anaesthetic agent.
3. Flow of contrast medium away from the needle tip and opacification of the joint space confirm adequate position. The joint capacity is about 10 ml.

With such criteria, the needle path is distant from the neurovascular bundle. Similar approaches can be used for ultrasound-guided arthrography of the hip [18].

### Knee

The knee joint is classically entered via a patellofemoral approach. Alternatively, an anterior approach targeting the anterior recess has been described more recently [19].

**Fig. 14:**
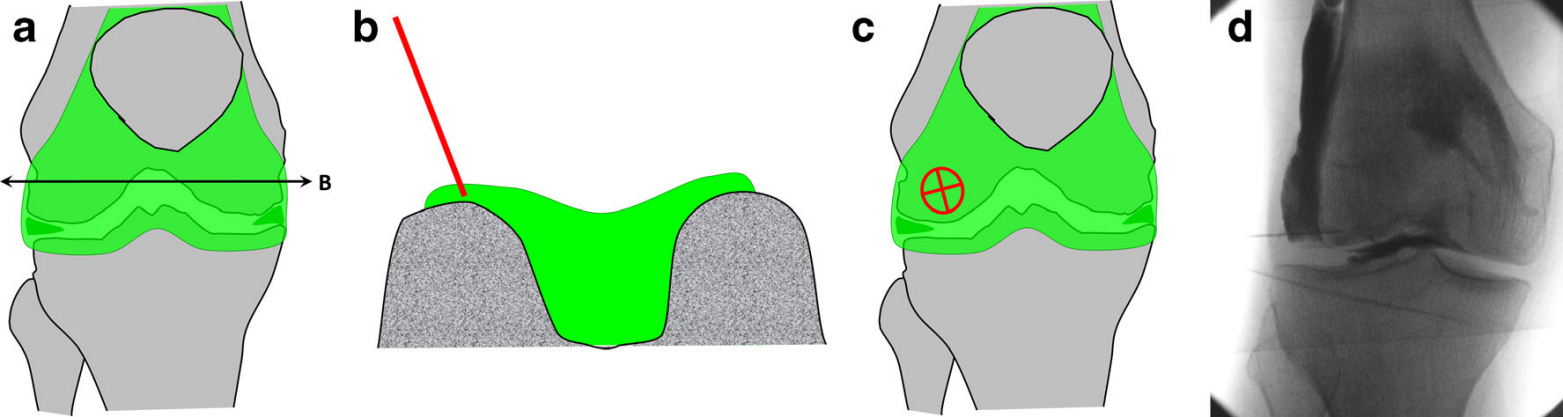
Anterior approach targeting the lateral femoral condyle as shown on a diagrammatic representation of the knee joint (**a**) with a transverse section through the femoral condyles (**b**). **c** The target is the anterior and lower aspect of the lateral femoral condyle. **d** The needle is inserted until bone contact and adequate position is confirmed by opacification of the joint space

For knee arthrography targeting the anterior recess (Fig. 14):

1. The knee of the patient is positioned in slight flexion. The target is the lower aspect of the lateral femoral condyle.
2. A 1.5-inch (3.8-cm) 22-gauge needle is inserted until bone contact and the injection is tested with an anaesthetic agent.
3. Flow of contrast medium away from the needle tip and opacification of the joint space confirm adequate position. The joint capacity is more than 40 ml but diagnostic arthrography is appropriately performed with a volume of 10-20 ml.

### Ankle

**Fig. 15:**
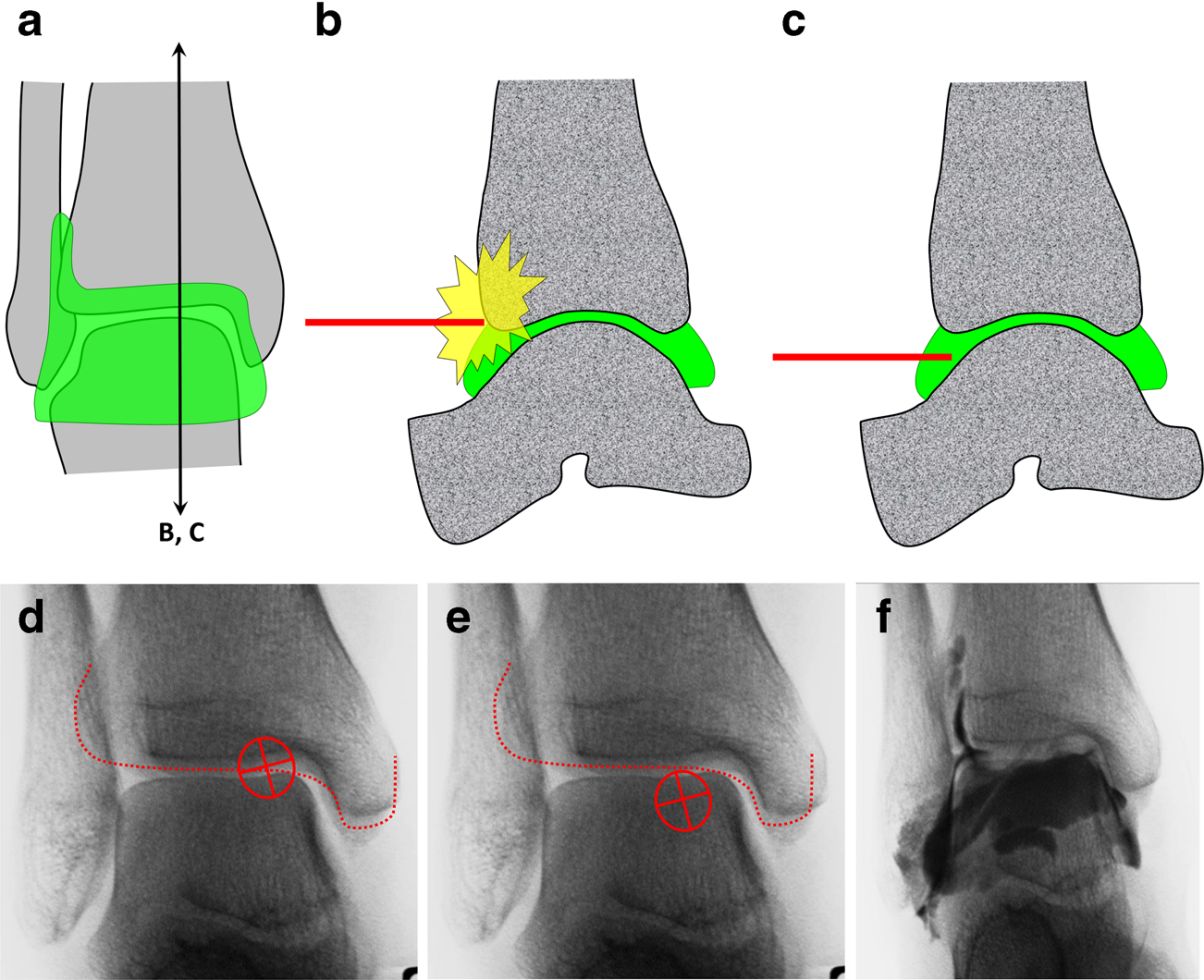
Diagrammatic representation of the ankle joint (**a**) with sagittal sections (**b**, **c**). There is a risk of hitting the anterior tibial margin (*red dotted line*) if the radiological joint space is targeted on an anteroposterior view (**d**). The anterior recess can be targeted just below the radiological joint space (**e**). After needle insertion, the adequate position is confirmed by opacification of the joint space (**f**)

When the radiological joint space of the ankle is targeted directly, there is a risk of hitting the anterior tibial margin. Instead, the anterior recess must be targeted just below the joint line. To do so, the following steps can be performed (Fig. 15):

1. The ankle is positioned in slight plantar flexion and the entry point is determined just lateral to the tibialis anterior tendon. The target is just below the joint space.
2. A 1.5-inch (3.8-cm) 25-gauge needle is inserted until bone contact and the injection is tested with an anaesthetic agent.
3. Flow of contrast medium away from the needle tip and opacification of the joint space confirm adequate position. The joint capacity is about 5 ml.

## Spine

### Cervical facet joints

**Fig. 16:**
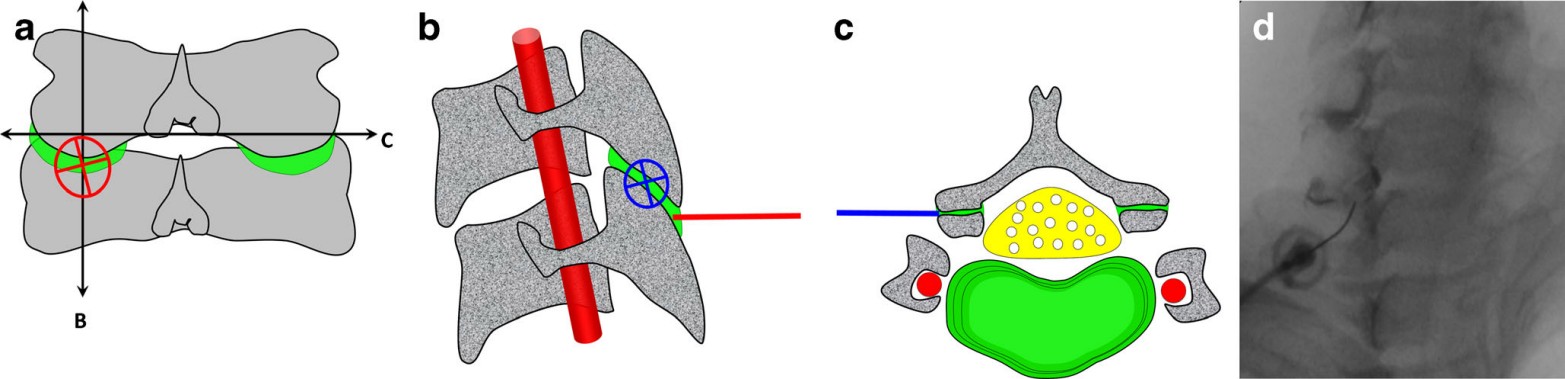
Diagrammatic representation of the cervical facet joints (**a**) with transverse (*B*) and sagittal sections (*C*). A lateral approach as represented in *blue* (**b**, **c**) may lead to perforation of major vessels and dura or nerve root sheaths. A posterior approach targeting the inferior recess as represented in *red* (**a**, **b**) may be safer because all major neurovascular structures are protected by the articular pillar. The needle is inserted until bone contact and the adequate position is confirmed by opacification of the joint space (**d**)

Classically, the cervical facet joints are accessed via a lateral, direct approach [20]. The major disadvantage of this technique is the risk of inadvertent perforation of major vessels and, more rarely, of neural structures [20]. A posterior approach targeting the articular recess may present a more effective strategy (Fig. 16). The technique is as follows:

1. The patient is positioned prone with the head rotated opposite to the injected side in order to avoid superimposition of the jaw on the cervical spine. The target is the inferior articular recess of the facet joint, which lies immediately below the most distal aspect of the inferior articular process. We caution against angulating the tube tangential to the facet joint in order to avoid passing through the joint and also to shorten the path.
2. A 3.5-inch (8.9-cm) 25-gauge needle is inserted until bone contact.
3. Flow of contrast media away from the needle tip in a horizontal direction with opacification of the joint space confirm adequate position. The joint capacity is about 1-2 ml.

This technique is also advantageous because bilateral injections can be performed without turning the patient and prepping the other side of the neck, as only the head has to be turned.

### Lumbar facet joints

Due to its curved orientation and frequent additional degenerative changes, the joint space is not easily entered via a posterolateral approach under fluoroscopy. It is easier to target the posterior and inferior recess [21]. This recess is larger when the physiological lordosis is reduced by placing a pillow under the patient’s abdomen.

**Fig. 17:**
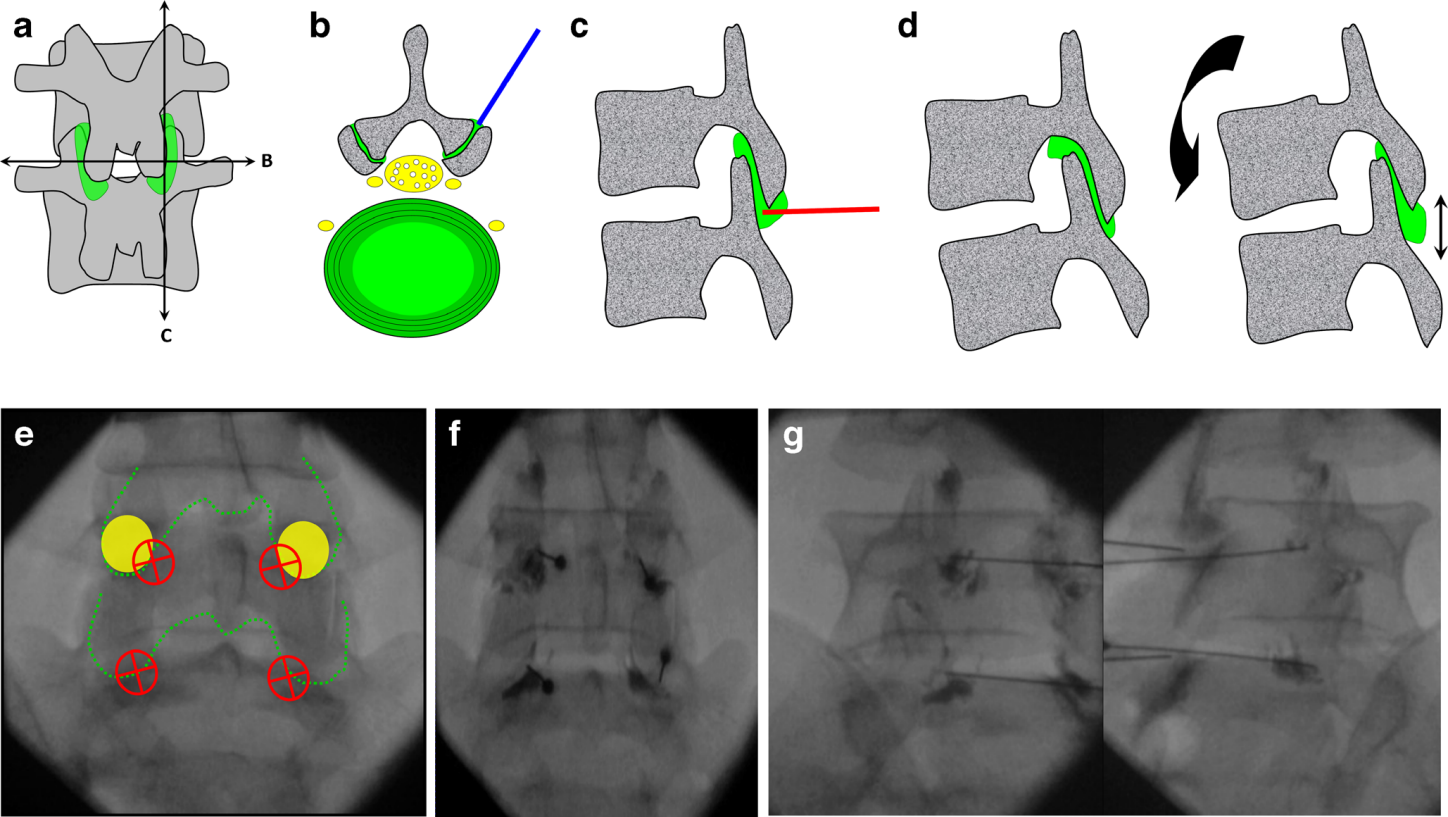
**a** Diagrammatic representation of the lumbar facet joints with transverse (*B*) and sagittal sections (*C*). **b** The classical posterolateral approach represented in *blue* can be impeded by the curved orientation and degenerative changes of the joint. **c** A posterior approach targeting the inferior recess may be an easier alternative. **d** The inferior recess can be enlarged by placing a pillow under the patient’s abdomen in order to reduce the physiological lordosis. **e** The target is the medial and inferior aspect of the pedicle projection (indicated by *yellow circles* at L5). **f** The needle is inserted until bone contact and the adequate position is confirmed by opacification of the joint space. **g** Oblique views are not required with this approach and are only displayed here for a better understanding of the anatomy

For lumbar facet arthrography (Fig. 17):

1. The target is the apex of the inferior articular process, which corresponds approximately to the medial and inferior aspect of the pedicle projection.
2. A 1.5-inch (3.8-cm) 22 or 25-gauge needle is inserted until bone contact.
3. Flow of contrast media away from the needle tip with opacification of the joint space in a typical ovoid shape confirm adequate position. The joint capacity is about 1-2 ml.

## Advantages and disadvantages of targeting the articular recess

The main theoretical advantage of the technique is that it facilitates articular injection when the joint space is obscured, either by patient positioning or degenerative changes to the joint. Moreover, reliable depth estimation can be provided by bone contact. By targeting the articular recess, the needle path is often shorter, thus diminishing the number of structures whose integrity is compromised. In our experience, this approach inflicts less pain to patients.

**Fig. 18:**
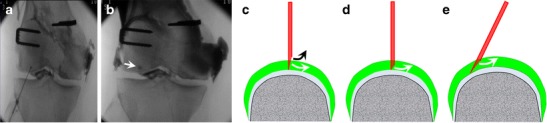
**a**, **b** This example of knee arthrography illustrates minimal pooling of contrast medium around the needle tip (*arrow*) reflecting mixed injection (intra-articular and extra-articular). **c** Mixed injection may occur when a shallow recess is approached perpendicularly with a long-bevelled needle. Better to use a short-bevelled needle (**d**) or to direct the needle tangentially to the cartilage (**e**)

A prerequisite of the technique is a precise anatomical knowledge of the articular recesses. Also, there is a risk of mixed injection where the contrast medium enters both the intra-articular and extra-articular spaces, especially in the absence of effusion or in the case of smaller joints (Fig. 18). Mixed injections usually occur when a shallow recess is approached perpendicularly with a long-bevelled needle. This risk is reduced by using a short-bevelled needle or by orienting the needle tangentially to the cartilage surface.

Ultrasound is used with a steadily growing frequency for guiding joint injections and aspirations as an alternative to fluoroscopy, mainly due to its lack of ionising radiation [22, 23]. We have observed that targeting the articular recess can be easily transposed to ultrasound-guided injections. With this technique, the needle should be kept parallel to the transducer to be visible, which makes targeting the recess rather than the joint line easier. A potential drawback of ultrasound-guided arthrography is the limited view of the whole joint and potential communications with neighbouring structures during injection.

## Conclusions

We reviewed some of the approaches most commonly performed for arthrography of the principal articulations. These approaches represent only a few of the numerous possibilities, and radiologists should feel free to tailor their own technique in order to safely perform arthrography.

By highlighting the capsular anatomy, we emphasised the approaches targeting the recess rather than the apparent joint space. This knowledge is also useful when performing ultrasound-guided arthrography, as capsular recesses are directly visible with this modality.
